# Correction: Identification of cytotoxic T cells and their T cell receptor sequences targeting COVID-19 using MHC class I-binding peptides

**DOI:** 10.1038/s10038-022-01024-1

**Published:** 2022-02-22

**Authors:** Tetsuro Hikichi, Michiko Sakamoto, Makiko Harada, Maki Saito, Yuka Yamane, Kimihisa Tokumura, Yusuke Nakamura

**Affiliations:** 1 OncoTherapy Science, Inc, Kawasaki, Kanagawa Japan; 2 Cancer Precision Medicine Center, Japanese Foundation for Cancer Research, Tokyo, Japan

**Keywords:** Cellular immunity, Sequencing

Correction to: *Journal of Human Genetics* 10.1038/s10038-022-01013-4, published online 2 February 2022

In this article the wrong table appeared as Table 1 only in article PDF; the table should have appeared as shown below.

**Table 1:** SARS-CoV-2-derived candidate peptides bind MHC class I (HLA-A*24:02, HLA-A*02:01, or HLA-A*02:06)

ID	Protein	Sequence	Length (amino acids)	Starting position	Affinity (nM)	HLA	Conserved in
P01	ORF1ab	TYASALWEI	9	4090	18	A*24:02	SARS-CoV
P02	S	PFAMQMAYRF	10	897	39	A*24:02	SARS-CoV
P03	ORF1ab	YDYLVSTQEF	10	3811	41	A*24:02	SARS-CoV
P04	ORF1ab	YYSQLMCQPI	10	2560	44	A*24:02	SARS-CoV
P05	ORF1ab	SYYSLLMPI	9	4628	46	A*24:02	SARS-CoV
P06	ORF1ab	AYANSVFNI	9	5080	56	A*24:02	SARS-CoV MERS-CoV
P07	ORF1ab	RYKLEGYAF	9	6676	62	A*24:02	SARS-CoV
P08	ORF1ab	FTYASALWEI	10	4089	69 21 5	A*24:02 A*02:01 A*02:06	SARS-CoV
P09	ORF1ab	SYSLFDMSKF	10	7039	85	A*24:02	SARS-CoV
P10	ORF1ab	RYFKYWDQTY	10	4677	90	A*24:02	SARS-CoV
P11	ORF1ab	TAYANSVFNI	10	5079	102	A*24:02	SARS-CoV MERS-CoV
P12	M	LWLLWPVTL	9	54	108	A*24:02	SARS-CoV
P13	ORF1ab	TYASALWEIQ	10	4090	111	A*24:02	SARS-CoV
P14	ORF1ab	YAYLRKHFSM	10	5138	115	A*24:02	SARS-CoV
P15	ORF1ab	SLFDMSKFPL	10	7041	6 18	A*02:01 A*02:06	SARS-CoV
P16	ORF1ab	LMYKGLPWNV	10	6077	7 8	A*02:01 A*02:06	SARS-CoV
P17	ORF1ab	FVLWAHGFEL	10	6108	8 13	A*02:01 A*02:06	SARS-CoV
P18	ORF1ab	YMPASWVMRI	10	3654	12 103	A*02:01 A*02:06	SARS-CoV
P19	ORF1ab	MMGFKMNYQV	10	5982	18 77	A*02:01 A*02:06	SARS-CoV
P20	ORF1ab	FELTSMKYFV	10	6115	18 59	A*02:01 A*02:06	SARS-CoV
P21	ORF1ab	MMILSDDAV	9	5147	35 13	A*02:01 A*02:06	SARS-CoV
P22	N	FGMSRIGMEV	10	315	37 105	A*02:01 A*02:06	SARS-CoV
P23	ORF1ab	TLMIERFVSL	10	5245	42 48	A*02:01 A*02:06	SARS-CoV
P24	ORF1ab	YVYNPFMIDV	10	6160	51 15	A*02:01 A*02:06	SARS-CoV
P25	ORF1ab	NVLAWLYAAV	10	3466	72 11	A*02:01 A*02:06	SARS-CoV
P26	ORF1ab	TLEPEYFNSV	10	5740	79 22	A*02:01 A*02:06	SARS-CoV
P27	ORF1ab	YSFLPGVYSV	10	3098	81 23	A*02:01 A*02:06	SARS-CoV
P28	ORF1ab	FMIDVQQWGF	10	6165	84 29	A*02:01 A*02:06	SARS-CoV

*S* spike protein, *M* membrane glycoprotein, *N* nucleocapsid phosphoprotein

